# Health literacy and health care experiences of migrant workers during the COVID-19 pandemic: a qualitative study

**DOI:** 10.1186/s12889-022-14487-w

**Published:** 2022-11-09

**Authors:** Soo Jin Kang, Ji An Hyung, Hae-Ra Han

**Affiliations:** 1 Department of Nursing, Daegu University, Daegu, South Korea; 2 Department of Nursing, Graduate School of Kyungpook National University, Daegu, South Korea; 3 School of Nursing, The Johns Hopkins University, Baltimore, MD USA

**Keywords:** Migrant worker, Pandemic, Qualitative research, Health literacy

## Abstract

**Background:**

Migrant workers are among the most vulnerable populations in society. This study explored the health-literacy experiences of migrant workers in South Korea and how the workers’daily lives have been affected by the coronavirus disease 2019 (COVID-19) pandemic.

**Methods:**

We conducted a series of semi-structured individual and focus-group interviews with 23 migrant workers (eight Cambodians, six Nepalese, four Sri Lankans, three Bangladeshis, and two Pakistanis) residing in the Daegu and Busan metropolitan areas of South Korea. All interviews were digitally recorded and transcribed verbatim. The data were analyzed using content analysis.

**Results:**

Migrant workers had difficulty accessing and using health care services due, in large part, to linguistic barriers and a lack of an adequate support system. Four main themes were identified: difficulty understanding and using medical services, obtaining necessary health and safety information, the impact of COVID-19, and protecting oneself from becoming infected with COVID-19. Most workers depended on information from social networking services (SNS) and co-workers.

**Conclusions:**

Migrant workers’ difficulty with health care access was exacerbated during the COVID-19 pandemic. The findings suggest the necessity of enhancing migrant workers' health literacy, along with the use of SNS as a viable pathway for sharing health information and resources.

## Background

Globally, there are 164 million migrant workers—people engaged in a remunerated activity in a state of which they are not nationals [1]—and their numbers are increasing [2]. While more than half of migrant workers (61%) are concentrated in higher-income countries, such as North America, the Arab States, and Europe, South Korea is one of the countries where there is a dramatic increase in the number of migrant workers. For example, before the 1990s, there were only approximately 50,000 migrant workers in South Korea [3]. Since the 1990s, South Korea has experienced a labor shortage in the manufacturing and construction industries, and the South Korean government has consequently allowed unskilled migrant workers to enter the country through its Employment Permit System (EPS). As of 2020, there were over 391,000 unskilled migrant workers in South Korea—mostly from China and Southeast Asian countries—accounting for 30.9% of its total foreign residents [4, 5].

Unskilled migrant workers are officially allowed to work in South Korea for a maximum of 4 years and 10 months, after which they must leave the country [6, 7]. The limitation on the permitted stay in South Korea means that such migrant workers may feel a need to maximize their earnings during their stay; the resulting pressure may increase the likelihood of their accepting jobs that could potentially expose them to physical and chemical hazards [8, 9]. Among migrant workers, poor working conditions increase the risk of workplace injuries and disease and lead to higher rates of adverse health outcomes, when compared with non-migrant workers [10, 11]. Nevertheless, because of their temporary residential status, unskilled migrant workers’ health and safety needs are often ignored [12], highlighting the vulnerability of this population in society.

The coronavirus disease 2019 (COVID-19) emerged in late 2019, and the subsequent pandemic has quickly become a serious global public health issue. As COVID-19 has spread worldwide, countries with high proportions of migrants have endeavored to implement strategies to prevent or to slow the arrival of new infections into the country and to aid recovery from the disease [13]. Given the novel nature of COVID-19, health and other relevant information regarding the pandemic continue to evolve at a rapid pace. Amidst this global crisis, migrant workers are more susceptible to the direct and indirect impacts of COVID-19 than native workers because of cultural and linguistic barriers and limited health literacy, as well as their frequent exclusion from policy protections [14]. In particular, the extensive new information on COVID-19 that is being produced can be overwhelming, especially for those with low health literacy [15].

In 2020, with the spread of COVID-19, the Korean government's approaches were to curb the spread of the virus by widespread virus screening tests, social distancing campaigns, and the nationwide mask mandate (which was combined with the country’s mask ‘rationing’ system). During the early stage of the COVID-19 outbreak, migrant workers faced many disadvantages against government policies. For example, the mask-rationing system excluded those without National Health Insurance or expired visas, and all foreigners were excluded from emergency disaster relief funds regardless of residency status. Additionally, migrant workers often experienced more reduced working hours than native workers, and it caused a decrease in wages [16, 17].

During public health crises, such as COVID-19, health literacy serves as an essential skillset to enable individuals to access relevant health information and services, to make appropriate decisions, and to practice healthy and preventive behaviors in their everyday lives. Despite the significant relevance of health literacy to health and health outcomes among migrant workers [18, 19], it is unclear how health literacy has affected migrant workers' experiences during the pandemic. To address this gap, this study aimed to explore health literacy experiences among migrant workers in South Korea and how health literacy influenced their experiences of the COVID-19 pandemic.

## Methods

### Design

We used qualitative approaches featuring both individual in-depth interviews and focus-group interviews.

### Setting and sample

We used snowball sampling to recruit participants from the Support Center for Migrant Workers in Daegu, South Korea. The inclusion criteria were as follows: (1) previously or currently employed through the EPS, (2) having lived in South Korea for at least 6 months, and (3) willing to provide informed consent to participate in this study.

### Data collection and procedures

For the purpose of this research, the study team designed a semi-structured interview guide (Table 1). The interview guide was developed based on four dimensions (i.e., understand, access, apply, and appraise) of health literacy [20] and comprised questions concerning health literacy experiences and how the COVID-19 pandemic had changed the participants’work and personal lives. Example questions included: “What has changed in your life as a result of the COVID-19 pandemic?”, “How do you usually receive health information?”, and “How are you obtaining information on COVID-19, and how are you using it to maximize your personal safety?” Interviews were conducted from June 20 to November 1, 2020, at which point data saturation (i.e., no additional themes identified [21]) was achieved.

**Table 1:** Topical areas with sample questions

Changes to work and personal life as a result of COVID-19	Question 1. Please tell us what has changed in your life as a result of the recent COVID-19 pandemic. In particular, please describe whether there have been any changes in your work, life (emotionally), and human relationships since the cluster outbreak of COVID-19 in Daegu in February, 2020
Access to and use of health information	Question 1. Please describe how you usually receive health information and the kind of information resources you are using (i.e., channels for obtaining health information; the kind of information you and your colleagues require; and events, encounters, and others that helped you to obtain the information you needed)
Understanding and evaluation of information	Question 1. Do you think that you can usually appropriately understand everything when you are provided with health-related information, go to a hospital, and/or meet with a medical professional?

*COVID-19* coronavirus disease 2019

To accommodate the national COVID-19 restrictions at the time of the study, we used a combination of individual and focus-group interviews that accorded with the quarantine guidelines, which included wearing a mask, washing hands, and maintaining social distance. We conducted 17 in-depth individual interviews and one focus-group interview with six migrant workers, totaling 23 participants. In-depth individual interviews were conducted in Korean or the participants preferred languages (in the latter case, an appropriate language interpreter was present to assist communication). The focus-group interview was conducted at the Support Center for Foreign Workers in Daegu, South Korea; the interview group comprised six Cambodians. The group discussion was conducted in Korean and Cambodian. To facilitate interactions among the focus group participants, we used vignettes based on health literacy experiences obtained from the in-depth individual interviews and existing literature [22, 23]. Interviews lasted 80 min on average (range = 40–100 min). All interviews were audio recorded and transcribed verbatim. During and immediately after each interview, field notes were taken in response to the vignettes and observations by the first author (SJ).

Each participant received approximately US $45 as a token of appreciation for their time. After the in-depth or focus interviews, if needed, follow-up contact through phone calls or text messages was made to confirm or to clarify questions about the comments made during the interviews. These follow-up calls were not audio-recorded.

### Ethical considerations

This study was approved by the Institutional Review Board of Yeungnam University College (YNC IRB/202006–15). All participants were informed of the purpose of the study. Written informed consent was obtained from every participant before the data collection. Participants were assured of anonymity and confidentiality of their information and the audio recordings of their interviews. Participants had the right to withdraw from the study at any time. All research methods were carried out in accordance with the relevant guidelines and regulations.

### Interview participants

Table 2 shows the characteristics of the participants. The sample included individuals from Cambodia, Nepal, Sri Lanka, Bangladesh, and Pakistan; 34.8% were Cambodian, and 26.1% were Nepalese. More than two thirds (69.6%) were male, 56.5% had completed high school, and the mean age was 31.7 (standard deviation = 6.0) years. More than half of the participants (52.2%) had lived in South Korea for 3–6 years. Regarding their Korean proficiency, only 43.5% were proficient in listening skills, with around 17.4% each reportedly being fluent in reading and speaking skills, respectively.

**Table 2:** Participants’ characteristics (*N* = 23)

Characteristics	Category		n	% or mean (SD)
**Sex**	Male		16	69.6
	Female		7	30.4
**Age (years)**	20–30		3	39.1
	31–40		8	52.2
	≥ 41		12	8.7
	Min–max		23–48	31.7 (6.0)
**Nationality**	Cambodia		8	34.8
	Nepal		6	26.1
	Sri Lanka		4	17.4
	Bangladesh		3	13.0
	Pakistan		2	8.7
**Education level**	≤ Middle school		4	17.4
	High school		13	56.5
	≥ College		6	6.1
**Occupation**	Unskilled job		20	87.0
	Engineer		3	13.0
**Total length of stay in Korea (years)**	< 3 3–6 7–10		3	13.0
			12	52.2
			8	34.8
**Visa status**	E-9		16	69.6
	E-7		4	17.4
	F-2		3	13.0
**Korean language proficiency**	Reading	Fluent	9	17.4
		Limited	14	82.6
	Listening	Fluent	10	43.5
		Limited	13	56.5
	Writing	Fluent	18	78.3
		Limited	5	21.7
	Speaking	Fluent	4	17.4
		Limited	19	82.6

E-9: Non-professional employment visa

E-7: Foreign nationals for working visa

F-2: Long-term residency visa

### Data analyses

All interviews were transcribed verbatim in the language used at the time of the interview. Transcripts made in other languages were translated into Korean for analysis. The first author checked all transcripts for accuracy by listening to the audio recordings and reviewing the field notes. Interview data were analyzed using a qualitative content analysis approach [2]. Content analysis is the approach to understanding the unknown phenomenon (e.g., health literacy experiences among migrant workers in South Korea) and providing knowledge [24]. For initial coding, two authors (SJ and NG) read each transcript independently and identified codes. Any discrepancies were resolved through group discussions until a consensus was reached. Methodological rigor was addressed by maximizing credibility and confirmability [25]. Credibility was assured through the validation and interpretation of study findings by an independent investigator with extensive knowledge and expertise in qualitative research, health literacy, and underserved populations. Confirmability was established through a series of group discussions until a consensus was reached and with the use of an audit trail. Related codes were grouped into themes and subthemes. Following a series of discussions among the study team members, themes and subthemes were finalized.

## Results

Table 3 summarizes the main themes and subthemes identified. There were four main themes: difficulty understanding and using medical services, obtaining necessary health and safety information, the impact of COVID-19, and protecting oneself from becoming infected with COVID-19. Each quote included in the text below is presented with a participant study code and the location of the quote for the participant’s interview transcript.

**Table 3:** Themes, subthemes, and sample quotes from the content analysis

Theme	Subtheme	Sample quotes
Difficulty understanding and using medical services in Korea	Limited Korean proficiency	*When I go to a clinic, the doctors often use the word “inflammation.” However, I do not understand what “inflammatory” means or what is wrong with me. I have only picked up a few words. I just say, “yes, yes, okay, I get it” [Participant K, 12]* *[Communication with] the doctor is very difficult. Some people do not visit clinics because they cannot explain [their problems] to the doctor [Participant J, 1]*
	Lack of knowledge of the Korean health-care system	It *is difficult to decide which doctor to see or which hospital to go to. When I go to a hospital, I [usually] have to go again the next week, and then the following week or month. I have to make several reservations, because I do not know which clinic is suitable for me or if I should go to a general hospital [Participant H, 13]* *Going to the hospital is the most complicated task in Korea. In my country, if I am sick, I just go to a general physician and get treated there, and if they cannot treat me there, they send me to another hospital. However, in Korea, I have to find out which [institution] to visit; thus, it is hard to understand it at first [Participant M, 10]*
	Insufficient time to visit a hospital	*When work ends, at nine, the dentist is closed. That is why we must go to the dentist during work hours [Participant L, 4]* *We only have Sundays off; however, the hospitals are closed on Sundays. Therefore, even if we are sick, we cannot go to the hospital unless it is really bad. That is why many people do not go to the hospital [Participant M,1]*
	Worries regarding fees	*Even if I do not need treatment, there is still a registration fee and bill. It is expensive for me [Participant G, 21]* *I was afraid that I would have to pay approximately 800,000–900,000 won for surgery. Everything is free in my home country, because of which I go back to my country to receive surgery [Participant L, 3]*
Obtaining necessary health and safety information	Limited health information available in one’s native language	*It is easier for us if they use English. I do not expect them to use Nepali, just English. I wish these things [information on COVID-19 symptoms] were presented in English [Participant B,18]* *When I go to hospital, I have an interpreter’s phone number; thus, we call and ask there. However, some hospitals say that they do not have one. I wish all of them had it [Participant G, 23]* *When I go to hospital, I bring an interpreter’s phone number with me; thus, I can call the interpreter and ask for help. Some hospitals have interpreters service over the phone, while others do not; I believe that all hospitals should have interpreters available [Participant I, 20].”*
	Relying on co-workers who speak Korean	*I am [usually] not very sure that I understand the [health-care professionals’ instructions]. I always check the information on the Internet and ask Korean people. I think that is the most important thing [Participant C, 13]* *When I had my first appointment with a doctor, I asked seniors [i.e., migrant workers who have been living in Korea for a long time] or Korean coworkers to accompany me; since then, most of the time my employer or his wife has accompanied me [Participant Q, 1]*
	Seeking opportunities or resources for learning Korean	*While speaking to a Korean person in my company when I was sick, I recognized my need to learn Korean to say the things I want to say [Participant C, 7]* *I use Korean portal a lot. There is a lot of information there. I want to speak Korean fluently; therefore, I try reading a lot of Korean [Participant D, 10]*
	Using technology to overcome limited language skills	*There is a text-translation application. It translates [text] that I copy and paste [into it] [Participant F, 8]* *If we do not understand something [at the clinic], the doctors look up the terminology on the internet using a cellphone, and we look it up on the internet as well. If we do not know what something means, we translate our question and show it to the doctor [Participant J, 16]*
Impact of COVID-19	Changes to daily life	*Work time has been reduced. In the past, it used to be 40 h a week; when [COVID-19] arrived, all factories ceased operation, because of which I could not work and there was no overtime. It was hard. Now, I do not work on weekends, and I do not work on my days off either [Participant B, 14]* *Before the pandemic, I could go outside to visit the foreign support center; however, I cannot go there now because of the risk of infection [Participant I, 8]*
	Witnessing discrimination	*The Korean government takes care of Koreans; however, I am just a foreigner. My country is poor, because of which we feel like we are not being taken care of by our own government, either [Participant B, 9]* *People have different views of foreigners, and at the time [of COVID-19’s early spread], I felt bad [because I experienced discrimination]. However, now I know that they could have had those feelings because of a fear of [COVID-19] [Participant C, 15]* *I had to get a COVID test, although the test center was far from here. I did not know how to go there. They did not provide me or my friends with transportation information. [Participant G, 7]*
	Psychological distress	*I can go back to my country; however, the airport is the most dangerous place because there are people there from all around the world. I thought that it would be worse if I caught the virus on the way because I would take it home to my family. I felt scared [Participant C, 1]* *I could not meet my friends because of [COVID-19], because of which I just worked alone, bought only essentials, and stayed at home. It was a hard time. I felt isolated [Participant P, 13]*
	Self-reflection	*Less work. I think that changed my life the most. After arriving here [in Korea], I tried to work out and work harder on weekends. I stay in the dormitory every weekend, although I wonder whether I can continue this work. When [COVID-19] first arrived and also now, [I think about] what I have done and [make plans] to protect myself [Participant B, 8]* *There is nothing we can do. Thus, I just [try to accept the situation and] think, “oh, this will not last long” [Participant J, 12]*
Protecting oneself from becoming infected with COVID-19	Adhering to preventive behaviors	*I am checking my body temperature and performing self-diagnosis daily [Participant N, 8]* *I travel to and from work alone, spray disinfectant on my body, keep my clothes separate [from other people’s clothes], and so on. And I always keep my mask on [Participant P, 8]*
	Belief in overcoming the virus	*I think that the [Korean] government has presented a strong prevent-and-control message to the people [that has helped] to reduce transmissions. The most important thing is that we overcome [COVID-19] [Participant F, 13]* *I believe I am safe here [in Korea] because we comply with the government’s quarantine guidelines. Thus, you have to believe that this is not going to last long [Participant J, 5]*
	Tracking positive cases	*There is an app for tracking the movement of confirmed COVID-19 patients. There is also an English-language Google Maps resource. We look at that. People translate it into our language and upload the information [onto SNS]. It is on Facebook [Participant A, 17]* *[We receive] patient-tracing [information] by text. Even if we are not good at speaking [Korean], we can all read the places [the patient] visited [Participant C, 3]*
	Learning about the disease	*I have been gathering a lot of [COVID-19 information] from Facebook. All of my friends do that, too [Participant D, 9]* *Since [COVID-19] is severe, they say, “do this” [hand sanitizing], “do that” [body temperature measurement]. However, there are also “do not do” things, such as [eating together]. I do not know much about [COVID-19] [Participant K, 7]*

*COVID-19* coronavirus disease 2019, *SNS* social network systems

### Theme 1: Difficulty understanding and using medical services

Interview participants had significant barriers to understanding how the South Korean health care system works and how to access necessary health care. We identified four relevant subthemes: limited Korean proficiency, lack of knowledge about the South Korean health care system, insufficient time, and concern about hospital fees.

Limited Korean proficiency was a common barrier to health care access. As per the national policy, migrant workers have to pass the Test of Proficiency in Korean (TOPIK) to be eligible for a job in South Korea. The TOPIK includes a basic Korean learning program as part of the TOPIK registration and test-taking processes. Some migrant workers chose to learn Korean through the TOPIK just to “get by” rather than spending money and time to learn the language. One worker described, “*Through the TOPIK, I only learned the basic [health-related] words […], such as ‘hands,’ ‘feet,’ ‘headache,’ or ‘abdominal pain.’ [Participant N, 1].”* After passing the Korean exam, most workers had to wait, per their description, an “indefinite” amount of time (at least 6 months) before entering South Korea; by the time they arrived in the country, they forgot the Korean vocabulary they used to know. A migrant worker expressed the challenges faced in communicating with a doctor: *“When I visited a clinic alone in South Korea, there was no one with whom I could communicate. I had to use gestures and only said, ‘I, pain’. [Participant E, 6]”* Language barriers resulted in unsatisfactory and poor patient-provider communication. Some workers reported that they did not receive sufficient information from health providers and consequently avoided them. One worker said*: “[The doctor] just said, ‘I know,’ although I do not think [the doctor] really understood. This made it uncomfortable for both the doctor and me [Participant 0, 7].”*

Lack of knowledge about the South Korean health care system made it even more challenging for the participants to use medical services. In South Korea, patients need to choose a primary or specialty doctor based on their signs and symptoms (as opposed to primary physician referrals to specialty doctors). Interview participants noted that the requirement to choose a doctor was confusing. One worker explained: *“It is hard for me to find the appropriate clinic or doctor by myself. I go to any clinic on the street, regardless of my problem. If it is the wrong clinic, they tell me where I must go [Participant A, 12].”* Another worker said, *“Generally, clinics provide a broad level of care. However, South Korean hospitals are too big and complicated; therefore, I need help from someone—going through the reception counter, picking up the prescription, and so on.. [Participant I, 10].”* As a result, many workers used complementary and alternative medicine, in accordance with their traditional cultures. One worker said *“Sometimes, we ask for medicine or purchase it from newly arrived friends or other migrant workers in South Korea [Participant R, 6].”*

Other significant challenges included lack of time and worries about service fees. Migrant workers preferred working extra hours to receive overtime pay; however, since most of the participants worked in small-sized businesses, their overtime or off-duty work was often unplanned and unpredictable. This resulted in a limited time window to schedule a doctor’s appointment for their health problems. Migrant workers felt more comfortable to make a clinic visit on weekends or at night than being excused during their work hours. Nonetheless, they were burdened by the higher cost of after-hour appointments: *“I must go to the nearest hospital because I need to make it quick. Our South Korean coworkers conveniently visit [hospitals], although we need to be sort of tactful in doing so. This is why I go see a doctor after hours or on weekends, although I need to pay more money for these visits [Participant T, 5].”*

After the COVID-19 outbreak in South Korea, migrant workers were dismissed from their work or had to take unpaid leave; hence, any cost related to medical service was perceived as a significant burden. One worker complained, *“Ever since the pandemic, few or no jobs have been available. I do not know when I will work. [However], I will be available when my boss clocks me in because I need money. Although the work has been sparse, I must [be on call and] wait until I am told to come in for work [Participant U, 15].”* All participants noted health insurance as an important determining factor to use health services. Migrant workers who were legally admitted to the country had health insurance; however, when their visas expired, they became undocumented and lost health insurance. Without health insurance, migrant workers avoided seeing a doctor, as it was simply too expensive. Even when workers had health insurance, returning to a home country was a better option for some who had to receive a surgery. One worker stated: *“I know that there is a long waiting time for surgery in my country. Health services in South Korea are good, although those in my own country are free or cheaper than here* [*Participant D, 12].”* Recently, most free health clinics for low-income migrant workers were closed due to the COVID-19 pandemic. One worker complained, *“I used to receive a free medical check-up. Ever since the pandemic, this service was all suspended; therefore, I do not have any place to go even when I get sick [Participant B, 22].”*

### Theme 2: Obtaining necessary health and safety information

Obtaining necessary health and safety information was another main theme, and it included three subthemes: limited health information in one’s native language, seeking opportunities or resources to learn Korean, and using technology to overcome limited language skills.

The participants had limited opportunities to acquire health information in South Korea. In particular, in the early stages of the COVID-19 pandemic (February–March 2020), the local government in Daegu provided real-time updates on COVID-19 cases, although the information was not available in a foreign language. One worker said, *“Every morning, a public safety alert message delivers on my phone, in Korean. Because the main target of these messages is South Koreans, people like us [foreign workers] cannot understand anything [Participant A, 7].”* Instead, migrant workers often relied on health information from family members, friends, and social networking services (SNS) back in their home countries. One worker reported: *“There is a famous and reliable family health encyclopedia in my home country [country name removed for confidentiality reasons]. When we have health problems in South Korea, we often access the encyclopedia by [calling home using] free internet calls [Participant S, 10].”*

Some workers were determined to learn Korean to better serve themselves. They searched for language-learning assistance from various sources, including foreign-worker support centers and senior migrant workers who could speak Korean well. One worker said, *“The Center offers a Korean language program, and many [friends] take classes there. It is free, too.”* Another worker said, *“I like it [the Korean class]. If one is absent too many times, the course requirement is not fulfilled. …before COVID-19, I used to work a lot on the weekends and I kept missing classes; therefore, I ended up dropping out of it [Korean class] [Participant 0, 28].”*

Participants discussed the use of technology to overcome the limited language skills*.* They heavily relied on translation or messenger apps on their smartphones. Before meeting with a doctor, some participants prepared what they would ask using a translation app: *“…I translate [what I want to say] before I go see my doctor, just to prepare myself [Participant F, 10].”* Another worker noted, *“I use a popular messenger app [Kakao Talk] to ask what I want to know. When I use the app widely used by South Koreans, it is easier to find someone who can give me a hand since many friends of friends [in Korea] also use the app.[Participant T, 16].”* Using the app, some Koreans even provided a simplified visual aid and migrant workers appreciated it: *“Oral pills, injection… such straightforward visuals help me understand what I should do [Participant J, 17].”*

### Theme 3: Impact of COVID-19

Four subthemes were identified in relation to the theme of the impact of COVID-19: changes in daily life, witnessing discrimination, psychological distress, and self-reflection. All participants experienced small or big changes in daily life ranging from limited or no social activities to economic precarity. One participant said: *“Since February 2020, my manager has completely banned us from going out on weekends. Over the last few months, I have spent my time in my dorm room, alone [Participant A, 26].”* Some noted reduced work hours and were worried about their ability to send money to their families. One worker said, *“The factory runs, although only few work there. …Fortunately, the factory has not closed. There have been days when I work only 2 h … The primary change for me is that the income has gone downward [Participant K, 15].”* In some cases, concerns were related to the fact that the pandemic made things more difficult for workers to adjust to the Korean society. For example, the centers for migrant workers were either closed or had changed the format of their education programs, including those for learning Korean and computer skills, from face-to-face to online. However, migrant workers rarely owned a computer and had to rely on their phones. Yet, a cellphone was not an appropriate learning device to engage in a 3-month course that required 3 h of study a day: *“…online education causes challenges for migrants […]; my friends gave up. I would prefer studying with other workers in a classroom [Participant U, 18].”*

Discrimination against migrant workers was also discussed. Participants mentioned feeling excluded from care as they did not receive timely information and care from the South Korean government. The majority of the participants reported feeling alienated as a result of being excluded from the Korean government’s COVID-19 emergency relief funds provided to its citizens. One worker said, *“The Korean government provides for its citizens. However, my country is poor and I am a foreigner. I do not feel that we are being taken care of…[Participant B, 9].”* They felt further discouraged by the Korean government’s decision to declare foreign workers as a “risk group” for the COVID-19 infection. One worker complained vigorously, stating: *“Why are we regarded as being confirmed cases? I even got a test [Participant M, 13].”* Another worker noted, *“I got the foreigner [COVID-19] test. None of my South Korean coworkers were tested. We work together; why was I the only one tested? Am I suspected of having [COVID-19] just because I am a foreigner? [Participant C, 5].”*

When participants discussed the impact of COVID-19, they mentioned that, during the initial outbreak of the virus in Daegu, they felt anxious and distressed because of the uncertain future. One worker reported, *“In February [2020], when [COVID-19] had just appeared, I wanted to return to my country. I was scared at that time. I could not go because there was no airline available [Participant S, 3].”* Another worker added that he felt like he might become depressed as a result of disconnection from the outside world: *“I was worried if I would contact COVID-19 back in February [2020]. I did not go outside often and even avoided taking the bus. I stayed away from other people as much as possible. I did not meet friends either. I rarely went grocery shopping [Participant I, 11].”*

Despite these negative emotions, some participants described engaging in self-reflection. The pandemic provided them a chance to think about what they were planning to do with their lives and how they can cope with distress from the pandemic. While money was of primary importance for migrant workers before the COVID-19 outbreak, the pandemic changed their perspectives. They recognized that their health and dream are most important. One worker, who had tested positive for COVID-19 and had stayed in a residential treatment center, said: *“The first quarantine facility was like a prison. I was confined in a room alone. I was intimidated and worried to be alone after the positive COVID-19 result… I prayed to Allah every day. I felt that I was being protected… I realized that I have put off countless important decisions in life. …It is a short life we live; I believe going after dreams even if one may fail is worthier than worrying and not giving it a try [Participant M, 18].”* Another worker said, *“I came to South Korea because I like K-pop. …These days, I realize that my life will not change other than earning more money with the current visa. I will take a [school] qualification exam when I go back to my country. I want to study at a South Korean college. Would not my life turn from a toilsome laborer to an engineer then? I want to become an engineer and live the rest of my life with confidence [Participant C, 20].”*

### Theme 4: Protecting oneself from becoming infected with COVID-19

The two subthemes related to the theme of protection from COVID-19 were: adhering to preventive behaviors and learning about the disease. Participants were worried about being exposed to the COVID-19 virus in their living and working environments. They tried to comply with the protective guidelines. One worker said, *“Our factory at first did not make any change when COVID-19 spread. …My boss said not to go around outside. Lately, there are hand sanitizers where we work and screen fences in the cafeteria. …My friends who work at different factories say there has been close care for COVID-19 prevention at their workplaces [Participant B, 13].”* Still, some were concerned about their workplace not necessarily implementing thorough protective measures: *“Every morning, my boss asks the foreign workers, including me, ‘Do you have a fever?’ But I do not have a thermometer, because of which I say ‘no.’ My boss never checks our temperature or prepares a thermometer for workers.”* Another participant said, *“I was never given a COVID-19 protection guide in my factory [Participant W, 6].”*

Participants said that learning about the disease was a way to prevent COVID-19. However, it was difficult for migrant workers to access rapidly changing new information about the pandemic because of the language barrier. At the time of our interviews, the Korean government had provided a free tracking app as a means to prevent further viral infection spread. However, this service was only provided in Korean. Consequently, the participants chose to consult Facebook to track positive cases: *“There are people on SNS who call themselves ‘reporters’ [Participant C, 21].” They translate the reports from Korean to other languages in real time. “We obtain information through them [Participant C, 21].”* In fact, most migrant workers sought information about COVID-19 from SNS, including Facebook and Twitter, and they believed the information they obtained from anonymous users—“reporters”—to be accurate and reliable. Collected information on COVID-19 was then shared with fellow migrant workers through SNS. Participants noted that posting translated reports on COVID-19 was almost occurring on a real-time basis (i.e., as soon as these reports become available) and that sharing information acquired through SNS was a way to express their concerns and care for one another.

## Discussion

This is one of the first studies to explore the health literacy experiences of migrant workers and how health literacy impacted their lives during the COVID-19 pandemic. The main themes included difficulty understanding and using medical services, obtaining necessary health information, the impact of COVID-19, and protecting oneself from becoming infected with COVID-19. Our analysis revealed that, for migrant workers, widespread barriers to health care existed even before the COVID-19 outbreak, indicating that these challenges are chronic and occur outside of extraordinary situations, such as pandemic events.

Most participants in the study reported difficulty understanding and communicating with health care providers due, in large part, to limited Korean proficiency. Approximately 50% of migrant workers in South Korea have limited Korean-language ability [8]. Insufficient Korean language proficiency leads to difficulties in understanding essential patient educational materials and medical forms that are written in Korean [26]. A recent national survey [27] revealed that one of the main reasons for unmet health care needs among immigrants in South Korea was communication difficulty (25.3%); the rate was much higher among non-professional employment visa holders, such as non-skilled migrant workers (54.2%). Migrant workers with low health literacy are at risk for significantly worse health outcomes than native workers [18, 19]. These findings suggest the need for more tailored language programs targeting non-skilled migrant workers. In particular, future language training programs need to include health literacy education and address key medical terminologies and orientation about the South Korean health care system.

We found that migrant workers’ vulnerability resulting from their limited health literacy was exacerbated during the COVID-19 pandemic. The interview participants noted insufficient knowledge of COVID-19 symptoms while mostly relying on coworkers or SNS to receive updates on COVID-19. It is interesting to note that the workers in this study found support from South Korean coworkers in the same workplace. This finding is consistent with that of a previous study, which reported that coworkers were the preferred health information source (31.6%) of migrant workers [8]. A possible reason is that their work permit visa (E-9) is based on allowance of employment at the workplace; therefore, the workplace is a primary channel for relationships with the South Korean society [28].

During the pandemic, the uncertain and limited information on COVID-19 may have increased the willingness of Korean coworkers to help foreign migrant workers who lack relevant sources of support. According to a recent analysis using COVID-19-related mortality data from 88 countries, countries with higher collectivist values had fewer COVID-19 deaths than those with high individualistic values, suggesting “caring for the community” as a dimension of social capital that is associated with public health [29]. Peer support may be a useful intervention strategy for migrant workers [30]. Therefore, to design migrant workers’ health programs, we suggest their workplace health program to include coworkers’ participation, rather than being based on an individual approach.

SNS was a common source of health information used by the migrant workers in the study. SNS can facilitate access to health information and encourage social support [31]. In a study of Korean-Chinese migrant women, network support through SNS messaging increased the participants’ exercise adherence [32]. Nevertheless, the presence of health claims made on social media is uncontrolled and often lacks monitoring for its validity. According to a recent analysis of over 2 million English tweets discussing a prescribed drug product (i.e., cannabidol for seizures), the approved condition ranked only 8 of 10 health claims made in association with this drug with 85% accuracy [33]. It would be important to better understand the quality of health information being shared on SNS and to identify effective strategies to communicate accurate and essential health information with underserved groups, such as migrant workers during the pandemic.

The migrant workers in the study experienced negative emotions resulting from the COVID-19 restrictions (e.g., central government’s mask rationing, government’s COVID-19 emergency relief funds, and administrative order mandating COVID-19 testing for migrant workers), as well discrimination against them especially during the first wave of COVID-19 (February–May 2020) [34]. Government policies, wherein Koreans were prioritized, severely excluded foreign residents, such as migrant workers. Perceived discrimination increases psychological distress and negatively affects physical and mental health [35]. Available national reports indicated that 20–30% of immigrants in Korea experienced discrimination [8, 27]. Migrant workers appear to experience more discrimination than the general immigrant population. For example, Choi and Song (2018) [36] reported that more than two thirds (77.2%) of migrant workers living in Gyeonggi province, Korea, experienced discrimination. Recently, Atteray et al. [37] reported the severity of mental health concerns among workers in Korea; from 2007 to April 2019, there were 170 deaths reported among Nepalese migrant workers, and 47 (27.6%) were by suicide. Korean governmental and non-governmental policies and programs have focused on addressing employment-related issues experienced by migrant workers (e.g., minimum or overdue wage, industrial accidents, and working hours) [38]. More attention should be paid to monitor migrant workers’ health outcomes while actively addressing the issue of discrimination against them. The workers who have signed a labor contract through the EPS are required to complete a preliminary training, which comprises Korean language education, understanding Korean culture and EPS, and industrial safety in their countries before departure [7]. We suggest that mental health screening and education needs to be included as part of the preliminary training in their countries.

### Limitations

We used convenience sampling to recruit the participants, which limits the generalizability of the study findings. For example, most study participants (87.0%) had lived in Korea for more than 3 years. Those with a shorter period of stay in the country might have different levels of exposure to the Korean health care system than those with longer years of stay. Additionally, we interviewed migrant workers between the first (February 18–May 5, 2020) and third waves (November 13, 2020–January 19, 2021) of the COVID-19 pandemic [34]. After the second wave of COVID-19, migrant workers began to receive COVID-related messages in English. Further, the administrative order to mandate COVID-19 testing for migrant workers was withdrawn because of concerns over human-rights violations. Despite these limitations, the study sample included migrant workers from various Asian countries. Most previous studies on migrant workers in Korea have focused on Korean-Chinese workers, the largest foreign population in Korea [39, 40]. Korean-Chinese workers, with an H-2 working permit visa, have an advantage in the labor market (e.g., no restriction of employment service and flexible employment contracts) over other Asian migrant workers (with an E-9 visa) in South Korea. Further, they tend to have a high level of Korean-language ability and a similar cultural background as native Koreans [28]. In contrast, migrants from South and Southeast Asia have very different backgrounds in terms of religion, culture, and language. Health inequality is prevalent among migrant workers according to the visa status in Korea [41], and further research is needed to understand these differences. Understanding these differences among migrant workers may be useful to develop strategies for increasing their right to health considering the diversity of migrant workers.

## Conclusions

In conclusion, this study provides a detailed description of migrant workers’ experiences with COVID-19. We found that migrant workers in Korea had difficulty accessing and using health care services, and this difficulty was exacerbated during the pandemic with considerably limited sources of health information. Their vulnerability mainly resulted from linguistic barriers and a lack of support system. Our findings highlight the need for a more tailored health literacy education program for migrant workers and workplace-based health programs with coworkers. Additionally, better health information communication system combined with mental health screening and education will be helpful to address inadequate or lack of health information sharing and mental health concerns in the migrant worker population.

## Data Availability

The datasets generated and/or analysed during the current study are not publicly available due to the risk of re-identification but are available from the first author on reasonable request.

## Abbreviations

COVID-19: Coronavirus disease 2019
EPS: Employment Permit System
SARS-CoV-2: Severe acute respiratory syndrome coronavirus 2
SNS: Social networking services
TOPIK: Test of Proficiency in Korean
